# Association of cardiovascular disease risk and blood biomarkers of Alzheimer's disease

**DOI:** 10.1002/alz70856_100656

**Published:** 2025-12-25

**Authors:** Grace Austin, Shaun Eslick, Jessica Ferguson, Manohar Garg, Ralph N Martins

**Affiliations:** ^1^ Macquarie University, Macquarie Park, NSW, Australia; ^2^ University of Newcastle, Callaghan, NSW, Australia; ^3^ Macquarie University, Sydney, NSW, Australia

## Abstract

**Background:**

Modifiable cardiovascular disease (CVD) risk factors such as dyslipidemia, type 2 diabetes T2D), hypertension, smoking, overweight and obesity contribute to the onset of Alzheimer's disease (AD). These risk factors may promote cognitive decline and AD pathology via vascular mechanisms such as small vessel disease. Few studies have examined the link between cardiovascular disease risk and plasma AD biomarkers in cognitively normal individuals. Therefore, this study aimed to determine the association between CVD risk and plasma AD biomarkers.

**Method:**

Secondary analysis of cross‐sectional data was conducted on 237 Australian adults, 30‐75yrs.

Predicted 5‐year and 10‐year CVD risk scores were calculated using the Australian CVD Risk Charts and Framingham Risk Equation, respectively. Measures included were age, sex, blood pressure, smoking and T2D status, high‐density lipoprotein, total cholesterol and the addition of medication use and history of atrial fibrillation for 5‐year risk calculations. Fasting blood was collected, and plasma separated by centrifugation and stored at ‐80°C. Plasma AD biomarkers Aβ1‐40, Aβ1‐42, GFAP, and NFL and pTau181 were measured using ultra‐sensitive Single Molecule Array (SIMOA) assay platform. Partial correlation analysis was performed between variables, adjusted for age and sex.

**Result:**

After adjustments, partial correlation analysis identified significant positive correlations between NFL and 5 year (r=0.22, *p* <0.001) and 10‐year cardiovascular risk (r=0.23, *p* <0.001). 5 year and 10‐year CVD risk was not significantly correlated with GFAP, Aβ42/40 ratio or pTau181. No significant correlation between cardiometabolic risk variables and AD biomarkers were observed.

**Conclusion:**

Findings suggest that higher predicted CVD risk was associated with higher expression of neurodegenerative biomarker NFL, independent of age and sex. This finding may indicate that CVDrisk factors may play a critical role in neurodegenerative pathways. Future studies that examine longitudinal associations and synergistic interactions between CVD risk factors and AD biomarkers are needed.

